# Investigating the COVID-19 related behaviors in the public transport system

**DOI:** 10.1186/s13690-021-00702-4

**Published:** 2021-10-22

**Authors:** Fatemeh Bakhtari Aghdam, Homayoun Sadeghi-Bazargani, Kavous Shahsavarinia, Fatemeh Jafari, Leila Jahangiry, Neda Gilani

**Affiliations:** 1 Road Traffic Injury Research Center, Tabriz University of Medical Sciences, Tabriz, Iran; 2 Tabriz Health Services Management Research Center, Department of Health Education and Promotion, Tabriz University of Medical Sciences, Tabriz, Iran; 3 Emergency Medicine Research Team, Tabriz University of Medical Sciences, Tabriz, Iran; 4 Department of Statistics and Epidemiology, Tabriz University of Medical Sciences, Tabriz, Iran

**Keywords:** Public transportation vehicles, COVID-19, Preventive behaviors

## Abstract

**Background:**

Determining people’s healthy behaviors related to COVID-19 could be effective in providing preventive measures. The present study aims to investigate preventive behaviors associated with COVID-19 including wearing masks and keeping physical distance among the passengers on buses and public taxis measures to evaluate the ventilation systems in these vehicles.

**Methods:**

This cross-sectional study was performed on 753 passengers on buses and taxis in Tabriz, northwestern Iran, from 15 February to 1 March 2021. Multistage sampling method was used to select the participants. Tabriz was socioeconomically divided into three areas, including high, moderate, and low socioeconomic status. Three researchers were observed passengers behaviors standing at the selected stations and assessed their behaviors according to study checklist.

**Results:**

In total, the data were gathered from 358 (47.5%) female and 395 (52.5%) male in public transport systems. The results of this study indicated that female passengers used masks significantly more than men (*P* < 0.001). About 40% of men and women did not keep a physical distance of at least one meter in the public transportation system. Failing to keep social distancing was mostly observed among people with low SES (*N* = 103, 54%) and those who were in city center (*N* = 88, 48.5%). According to the multivariate regression analysis, for not wearing mask: being male [OR 4.94; 95%CI (2.62–9.28)] and using bus [(OR 3.27, 95%CI (1.73,6.19)], and for not physical distancing: having age under 25 years [OR 2.58; 95%CI (1.53,4.36)] and low socioeconomic status (SES) [OR 5.19; 95%CI (3.25,8.30)], and for no ventilation: using bus [OR 1.57; 95%CI (1.05,2.34)] were significant predictors.

**Conclusion:**

Our results suggest that gender, type of vehicle, age, and SES were significant predictors of non-adherence to COVID-19 preventive behaviors in public transport during the pandemic. Given that social distancing is less observed in the public transportation system in Tabriz, Iran, it is necessary for government to consider and monitor guidelines to protect the passengers from COVID-19 infections by providing measures to maintain physical distance in public transportation systems. It may be possible to force vehicle owners who do not comply with health protocols to keep some distance by imposing fines.

## Background

The World Health Organization (WHO) has declared that COVID-19 outbreak is a global emergency and international concern since January 2020 [1]. The first cases of COVID-19 were reported in Iran in February 2019 [2] and the number of confirmed cases reached 479,825 with more than 36,000 deaths in November 2020 [3]. Studies have shown that keeping physical distance, reducing overcrowding, improving ventilation, and using masks can effectively reduce the spread of the disease [1, 4]. The preventive measures in public and indoor environments greatly contribute in reducing the disease transmission. Public transportation systems accelerate the spread of respiratory diseases such as influenza and coronaviruses worldwide due to high occupant density in an enclosed space [5, 6].

According to the international association of public transportation (UITP), public transportation system is among the most important sectors affected by COVID-19. At the beginning of the pandemic, public transportation systems reduced their performance by 80–90% in major cities in China, Iran, and the U.S., and by 70% in the U.K. [7].

Based on the government reports, the number of trips was decreased when people was asked to stay home and don’t travel unless it’s absolutely necessary to prevent the spread of COVID-19 [8, 9]. Several studies have indicated a statistically significant relationship between acute respiratory infections such as COVID-19 and using bus before 5 days of symptom onset [10] and found that public transportation system plays an important role in developing COVID-19 [4, 11].

There are strong evidences that show virus can also be transmitted through the air, therefore ventilation is an important factor in reducing COVID-19 indoors. Close contact is the most common way of transmitting the infection. However, airborne transmission has also been reported under certain conditions such as being indoors with poor ventilation for a prolonged period of time [2, 3]. Zheng et al. demonstrated that improved ventilation may reduce the risk of virus transmission in the public transportation system [4]. Vuorinen et al. (2020) emphasized that reducing crowding in the public transportation systems, improving their ventilation, and increasing the physical distance, particularly have an important role to prevent spreading of COVID-19 pandemic in public transport systems [2]. Ventilation and surface disinfection of transportation systems have been reported to be the effective factors in reducing pollutants in electric buses in Italy [5]. In some regions, there were certain restricting rules in the public transportation system, such as limiting the ridership to 15 passengers per city bus and 30 per rail car [6]. In the U.K., maintaining a distance of two meter has been considered necessary in public transport systems [2].

UITP considers maintaining high levels of services to ensure safe distancing among the main challenges in public transport systems. There are some guidelines for using public transport services including cleaning vehicles and surfaces frequently, checking the fever of staff and passengers, improving vehicle ventilation, using masks, and keeping physical distance [2].

At the beginning of COVID-19 outbreak in Iran, a large part of public transportation systems was eliminated, but resumed the operations after a while. Tabriz as the capital of East Azerbaijan province is the third most populous largest city of Iran with approximately 1,600,000 inhabitants. Like the other cities public transportation system in Iran, there was significant decrease in the number of their passengers during the COVID-19 pandemic. Since public transport is important for daily work travel of people, it is not possible to be completely shut down in the pandemic situation. The system had to adapt preventive measures related to COVID-19 pandemic guidelines [8].

The aim of this study is to investigate people’s coping behaviors related to COVID-19 in public transport systems in Iran. Studies have suggested that determining people’s healthy behaviors regarding COVID-19 could be effective in providing preventive measures [7].

Due to the high prevalence of COVID-19 and the importance of detecting preventive behaviors for reducing the incidence of the disease [7], the present study aims to investigate preventive behaviors associated with COVID-19 including wearing masks and keeping physical distance among the passengers on buses and public taxis measures to evaluate the ventilation systems in these vehicles.

## Methods

### Study design and participant recruitments

This cross-sectional study was performed on 753 passengers on buses and taxis in Tabriz, northwestern Iran, from 15 February to 1 March 2021. Multistage sampling method was used to select the participants. Tabriz was socioeconomically divided into three areas, including high, moderate, and low socioeconomic status, each of which areas were considered as the clusters. Bazaar, which was a crowded area in city center and has transportation service to almost all the regions, was considered as a separate area for data sampling. One area was randomly selected from each cluster as the study area. Rahnemaei station, Abresan station, and Railway station were selected from clusters with high, moderate, and low socioeconomic statuses (SESs), respectively. Samples were selected using convenience sampling method. In total, three researchers were observed passengers behaviors standing at the selected stations and assessed their behaviors according to study checklist. The first researcher was trained to observe and record COVID-19 related behaviors of the passengers in the two front seats, the second researcher was trained to observe and record COVID-19 related behaviors of the passengers in the middle two seats, and the third researcher was trained to observe and record COVID-19 related behaviors of the passengers in the last 2 seats of the randomly selected buses. All researchers also recorded the behavior of those passengers who were standing in the aisle related to their area and evaluated ventilation status of the buses and taxis considering if the window was fully or partially open in their defined area.

Observations were made on working days at 07:30 to 08:30 AM, and 13:30 to14:30 PM, and 17:00–18:00 PM, during which people spent time commuting between home and work and there was more traffic, as well as an off day. Data were collected from 753 people.

The sample size was calculated using the formula: *n* = Z^2^P (1-P)/d^2^, when Z is 1.96, *P* = 0.5 (assuming that 50% of people would either be engaged in preventive behaviors related to COVID-19), d = 0.05, and 95% confidence interval to be 400 people [8]. However, based on the effect size in cluster sampling, the final sample size was calculated as 753 people. Considering the ratio of three passengers on the bus to two passengers on the taxi, 460 passengers on the bus and 233 passengers on the taxi were included.

### Inclusion and exclusion criteria

Those taxis and buses on the research areas were selected to be included in the study that was visible to the researchers.

Data were collected using a checklist that was developed based on a literature review and consist of age (approximate), gender of the passengers, area (Bazaar, high SES region, moderate SES region, and low SES region), and type of the vehicle (taxi/bus), keeping physical distance, wearing masks by the passengers and drivers, opening windows to help ventilation and air circulation in the vehicles.

The preventive behavior for keeping physical distance on the taxi was considered if there were one passenger in the front seat and two passengers in the back seats, which was recorded by “Yes” or “No”. The preventive behavior for keeping physical distance on the bus was considered if every second seats were left empty and there was at least one meter distance between the passengers. Wearing mask by the passengers was another preventive behavior that researchers had recorded on the vehicles. Opening at least a window in the taxi and three windows in the bus were the next preventive behavior related to COVID-19 in public transportation systems. The content validity of the check list was qualitatively assessed by an expert panel including 11 specialists in the field of traffic, health education, epidemiologist and psychologist. The responses from the expert panel were used to alter and modify some of the items. The scores of content validity index (CVI) were computed on the basis of the simplicity, clarification, and relevancy of each item. A CVI score higher than 0.75 was considered as reasonable [11]. Content validity ratio (CVR) was, also, calculated based on the necessity and importance of each item. CVRs equal to or higher than 0.59 were considered as good content validity [11]. The CVI and CVR for the scale were 0.85 and 0.77, respectively. The internal consistency reliability of the scale was assured by calculating Cronbach’s alpha (α = .7). Spearman–Brown coefficient was used to assess the stability of the scale over time (*r* = .72).

### Data analysis

Statistical analyses were performed using the Statistical Package for Social Science, Version 18, for Windows (SPSS Inc., Chicago, IL, USA). The normality of the data was analyzed using a Kolmogorov-Smirnov test and the normal distribution of data was confirmed. The characteristics of the participants were summarized as numbers, percentages, or means with standard deviations, where appropriate. t-tests, and chi-square were used to compare the study sub-groups. In all tests, a value of *p* < 0.05 was considered statistically significant. Univariate and multivariate logistic regression were calculated to predict the behavior of not wearing a mask, not having a suitable distance and closing the window on the variables of gender, age, economic status and type of vehicle. In order to evaluate the multivariable logistic regressions, we used Receiver Operating Characteristic (ROC) curves and correctly classified values. The closer these values are to 1 hundred (in correctly classified value) and one (in ROC curve), the better the model fits.

## Results

In total, the data were gathered from 358 (47.5%) female and 395 (52.5%) male in public transport systems. Of the 753 passengers, 678 (90%) used masks. Of these 432(87.4%) were observed in the buses and 246 (95%) were reordered in the taxies, while 14 women (4%) and 61 men (15.5%) did not wear masks, indicating that female passengers used masks significantly more than men passengers (*P* < 0.001). There was no significant difference between men and women in terms of keeping the physical distance. Our results showed that more than 40% of men and women did not keep physical distance of at least one meter in the public transportation system.

According to our results, 209 passengers (28%) were in the approximate age group under 25 years old, 25 (12%) of them did not wear masks and 85 (41%) did not keep safe physical distancing. Also, 412 (55%) passengers were in the approximate age group of 26 to 50 years old, 30 (7.5%) passengers did not wear masks (Table 1). By analyzing gender in different age groups, the results showed that the group over 50 years old used masks significantly less than other groups and approximately 15% of people in this group did not wear masks (Table 1).

**Table 1:** Behaviors of wearing a mask and keeping physical distance based on age and gender in public transport system in pandemic of COVID-19 (Iran, 2021)

		Women N (%)		Men N (%)		Total	
**Facemask use**	Age years	Yes	No	Yes	No	Yes	No
	< 25	97 (96.0)	4 (4.3)	87 (80.6)	21 (19.4)	184 (88.0)	25 (12.0)
	26–50	215 (96.0)	9 (4.0)	167 (88.8)	21 (11.2)	382 (92.7)	30 (7.3)
	> 50	32 (97.0)	1 (3.0)	80 (80.8)	19 (19.2)	112 (84.8)	20 (15.2)
	Total	344 (96.1)	1 (3.0)	334 (84.6)	61 (15.4)	678 (90.0)	75 (10.0)
	*P*-value	0.96		0.08		0.01	
**Physical distancing**	Age years						
	< 25	63 (62.4)	38 (37.6)	61 (56.5)	47 (43.5)	124 (59.3)	85 (40.7)
	26–50	132 (58.9)	92 (41.1)	130 (69.1)	58 (30.9)	262 (63.3)	150 (36.4)
	> 50	29 (87.9)	4 (12.1)	71 (27.1)	28 (28.3)	100 (75.8)	32 (24.2)
	Total	224 (62.6)	134 (37.4)	262 (66.3)	133 (33.7)	486 (64.5)	267 (35.5)
	*P*-value	0.006		0.03		0.007	

Table 2 shows COVID-19 related preventive behaviors according to SES level. Preventive behaviors were gathered of 182 (24%) passengers from city center, 192 (25.5%) passengers from low SES, 190 (25.1%) passengers from moderate SES, and 189 (25%) passengers from high SES. In comparison to other area, the passengers in the low SES level were more likely to not wear a mask (*N* = 26, 13.5%). Moreover, failing to keep social distancing was mostly observed among people with low SES (*N* = 103, 54%) and those who were in city center (*N* = 88, 48.5%).

**Table 2:** Behaviors of wearing a mask and keeping physical distance based on gender and location in public transport system in pandemic of COVID-19 (Iran, 2021)

		Women N (%)		Men N (%)		Total	
**Face mask use**	**Location**	Yes	No	Yes	No	Yes	No
	City center	120 (96.0)	5 (4.0)	49 (86.0)	8 (14.0)	169 (92.9)	13 (7.1)
	Low SES	66 (98.5)	1 (1.5)	100 (80.0)	25 (20.0)	166 (86.5)	26 (13.5)
	Moderate SES	76 (90.5)	8 (9.5)	97 (91.5)	9 (8.5)	173 (91.1)	17 (8.9)
	High SES	82 (100.0)	0 (0.0)	88 (82.2)	19 (17.8)	170 (89.9)	19 (10.1)
	Total	344 (96.1)	14 (3.9)	344 (84.6)	61 (15.4)	678 (90.0)	75 (10.0)
	*P*-value	0.01		0.09		0.20	
**Physical distancing**	City center	61 (48.8)	64 (51.2)	33 (57.9)	24 (42.1)	94 (51.6)	88 (48.5)
	Low SES	34 (50.7)	33 (49.3)	55 (44.0)	70 (56.0)	89 (46.4)	103 (53.6)
	Moderate SES	61 (72.6)	23 (27.4)	89 (84)	17 (16)	150 (78.9)	40 (21.1)
	High SES	68 (82.9)	14 (17.1)	85 (79.4)	22 (20.6)	153 (81.0)	36 (19.0)
	Total	244 (62.6)	134 (37.4)	262 (66.3)	133 (33.7)	486 (64.5)	267 (35.5)
	*P*-value	< 0.001		< 0.001		< 0.001	

Based on Chi Square physical distancing was not maintained by the passengers in both vehicles (Table 2). In total, 294 taxis and buses were examined, 229 of which were properly ventilated. Out of 134 buses, 18 buses and out of 160 taxis, 47 taxis did not have appropriate ventilation (Table 3).

**Table 3:** Mask wearing behaviors, keeping physical distance, and ventilation status in public transportation system in pandemic of COVID-19 (Iran, 2021)

	Bus N (%)		Taxi N (%)		*P*-value
	Yes	No	Yes	No	
Face mask use	432 (87.4)	62 (12.6)	246 (95)	13 (5)	0.001
Physical distance	323 (65.4)	171 (34.6)	163 (62.9)	96 (37.1)	0.50
Ventilation	116 (86.56)	18 (13.43)	113 (70.62)	47 (29.37)	0.03

Table 4 presents the results of univariate and multivariate modeling, including odds ratios and their corresponding 95% confidence interval (CI) for the association between the behavior of not wearing a mask, not having a suitable distance and no ventilation and the variables of gender, age, economic status and type of vehicle. According to the multivariate regression analysis, for not wearing mask: being male [OR 4.94; 95%CI (2.62–9.28)] and using bus [(OR 3.27, 95%CI (1.73,6.19)], and for not physical distancing: having age under 25 years [OR 2.58; 95%CI (1.53,4.36)] and low socioeconomic status (SES) [OR 5.19; 95%CI (3.25,8.30)], and for no ventilation: using bus [OR 1.57; 95%CI (1.05,2.34)] were significant predictors. Also, People under the age of 25 were 2.58 times more likely to miss the distance than those over the age of 50 years. While among people 26–50 years old (*P* < 0.001), the odds of physical distancing was equal to 1.78 times compared to people over 50 years old (*P* = 0.021). Univariate analysis showed that Unmasked people on the bus were 2.73 times more likely than taxis (P < 0.001). Non-observance of distance also showed a decreasing trend with increasing SES (*P* < 0.05). The odds of no ventilation in bus was 1.59 times compared to taxi vehicle (*P* = 0.025).

**Table 4:** Binary logistic regression model of association of sex, age, location and vehicle with not mask wearing, not physical distancing

	Not wearing a mask				not physical distancing				No Ventilation			
	Univariate OR (95% CI)	P	Multivariable OR^*^ (95% CI)	P*	Univariate OR (95% CI)	P	Multivariable OR^*^ (95% CI)	P**	Univariate OR (95% CI)	P	Multivariable OR^*^ (95% CI)	P***
**Gender**												
Male	4.49 (2.46,8.18)	< 0.001	4.94 (2.62,9.28)	< 0.001	Ref.		Ref.		Ref.		Ref.	
Female	Ref		Ref		1.18 (0.87,1.60)	0.282	1.04 (0.74,1.47)	0.819	1.37 (0.97,1.94)	0.077	1.22 (0.84,1.77)	0.292
**Age**												
< 25	0.76 (0.40,1.43)	0.944	1.07 (0.55,2.09)	0.829	2.14 (1.32,3.48)	0.002	2.58 (1.53,4.36)	< 0.001	1.49 (0.86,2.58)	0.156	1.41 (0.80,2.47)	0.231
26–50	0.43 (0.24,0.80)	0.008	0.76 (0.40,1.43)	0.389	0.43 (0.24,0.80)	0.008	1.78 (1.09,2.90)	0.021	1.31 (0.79,2.17)	0.303	1.30 (0.76,2.20)	0.336
50>	Ref.	Ref.			Ref.		Ref.		Ref.	Ref.		
**Location**												
City center	Ref.		Ref.		3.98 (2.50,6.33)	< 0.001	4.07 (2.52,6.59)	< 0.001	1.40 (0.84,2.34)	0.196	1.29 (0.76,2.19)	0.344
Low SES	2.04 (1.01,4.10)	0.046	1.28 (0.61,2.70)	0.516	4.92 (3.10,7.80)	< 0.001	5.19 (3.25,8.30)	< 0.001	1.33 (0.80,2.22)	0.267	1.31 (0.79,2.20)	0.299
Moderate SES	1.28 (0.60,2.71)	0.524	0.87 (0.40,1.92)	0.739	1.13 (0.69,1.88)	0.626	1.10 (0.66,1.82)	0.721	1.58 (0.96,2.61)	0.071	1.54 (0.93,2.54)	0.095
High SES	1.45 (0.70,3.04)	0.320	0.94 (0.43,2.04)	0.879	Ref.		Ref.		Ref.	Ref		
vehicle												
**Bus**	2.73 (1.46,5.04)	0.002	3.27 (1.73,6.19)	< 0.001	1.11 (0.81,1.52)	0.504	1.14 (0.81,1.61)	0.449	1.59 (1.08,2.35)	0.018	1.57 (1.05,2.34)	0.025
**Taxi**	Ref.	Ref.			Ref.	Ref.			Ref.		Ref.	

*Note*: * Correctly classified = 90.04% and ROC curve = 0, ** Correctly classified = 90.04% and ROC curve = 0.76 for multivariable logistic regression model, *** Correctly classified = 78.49% and ROC curve = 0.71 for multivariable logistic regression model .76 for multivariable logistic regression model

## Discussion

The present study aimed to investigate COVID-19 preventive behaviors in the public transportation systems. The results showed that nearly half of the people did not keep the physical distance of one meter. Consistent with our results, Betkier and et.al investigated city residents to determine their current transport safety-related preferences. They showed that only 53.6% of the participants maintained physical distancing in the public transportation systems [12].

WHO has recommended that maintaining an interpersonal distance of 1.5–2 m minimize the risk of nose and mouth droplets transmission [13]. A review study also showed when people are one meter or more apart from each other, the probability of transmitting the disease is much lower than the time their physical distance is less than one meter. The further the physical distance among people, the more protection against the disease would be. For every one meter of distance, the probability of protection against the disease is doubled. However, various studies have recommended maintaining a distance of two meter or more [13, 14].

Some studies have indicated that the risk of developing the disease was reduced by 82% by maintain a distance of one meter or more. Keeping a distance has been known from the past as an effective factor for preventing infectious disease transmission and mortality [15, 16].

Given that social distancing is less observed in the public transportation system in Tabriz, Iran, it is necessary for government to consider and monitor guidelines to protect the passengers from COVID-19 infections by providing measures to maintain physical distance in public transportation systems. It may be possible to force vehicle owners who do not comply with health protocols to keep some distance by imposing fines. Also, policy makers and decision makers can design and implement policies that have been applied in other countries’ public transport systems to maintain physical distancing between individuals; For example, in some regions, the ridership in the public transport system has been limited to 15 passengers per bus [17]. In the U.K., maintaining a distance of two meter has been considered necessary in public transportation systems [2].

Keeping social distancing and limiting close contact are considered the most effective approach for COVID-19 prevention, because people with mild or asymptomatic disease can also pass and transmit the virus to others [18]. COVID-19 symptoms appear after a median incubation period of approximately 5 days, with a range of 2–10 days [19] and virus can be transmitted during this period [19, 20]. Since people can infect others during the incubation period, the possibility of disease transmission is accelerated if the physical distancing is not observed in the public transport system. Therefore, it may be possible to reduce contacts and disease transmission by designing and implementing appropriate policies. Moreover, providing face masks and hand sanitizers are the recommended strong measures to be used in public transportation system in comply with maintaining physical distancing.

Passengers in Tabriz as a city in a developing country; prefer to use public transport for their daily activities and many of those have to continue commuting rely on public transport system. At the beginning of the COVID-19 pandemic, Tabriz like other cities in Iran have had to impose massive restrictions on public transport system [2, 21], but during the third wave of the pandemic, using the public transport significantly increased because of their convenient, comfortable access to the urban area, and lower prices compared to private cars [22]. It is recommended that public transport system officials should increase the system capacity during the COVID-19 pandemic and make them COVID-safe for riders and passengers. Also, appropriate policies should be adopted to maintain physical distancing in the public transport system and necessary training should be provided to the staff and passengers.

In the present study, 90% of the passengers used masks, with lower proportion among men than women. A similar study reported that 80.4% of the passengers used masks [10]. Considering that public transportation is an enclosed space, wearing a mask should be mandatory for all the passengers. Studies have shown that the mandatory mask-wearing policy was associated with the reduced risk of COVID-19 morbidity and decrease the infection risk by 85% [22]. Masks act as a barrier that prevents the polluted air exhaled by patients or carriers from being inhaled by healthy people. Therefore, wearing face masks plays an important role in reducing the disease prevalence [23]. The results of the present study indicated that more than 30% of the men did not wear masks and over 50% of the passengers not have adherence to keep physical distance in city center and low SES region. Also, more than 40% of the vehicles did not have proper ventilation. These results warned of the widespread outbreak of COVID-19 in enclosed public spaces, which highlighted the importance of taking precautions and providing training. As the weather was cold at the time of the study, the windows were mostly closed on the vehicles and ventilation was not provided. It seems that poor ventilation was the most reasons for high transmission of COVID-19 in public transport systems [2, 24]. Therefore, decision makers should take fundamental steps to improve ventilation.

According to the taxi association, since COVID-19 outbreak, it was decided that two passengers should sit in the back seats and one passenger in the front seat; the cost of the vacant seat be divided between the three passengers. In low SES regions, most of the passengers did not accept to pay extra to maintain physical distancing. Therefore, the taxi driver picked up four passengers and, consequently, the physical distance was not practically observed. In low SES regions, some passengers may not be able to pay for masks. Therefore, transportation officials should make plans to distribute masks at low price, especially in city center and low SES regions.

In terms of socio-demographic factors related to mask wearing, physical distancing, and proper ventilation, our results suggest that gender and type of vehicle for mask wearing, age and location for physical distancing, and type of vehicle were significant predictors of preventive behaviors in public transport. Consistent with our results, Abdullah et al. in a similar study found that gender, car ownership, and employment status were significant predictors of traveling during COVID-19 pandemic [25]. It seems that socio economic factors such as age, socio economic status were important predictor in adoption of COVID-19 preventive behavior in public setting [26]. In fact, social factors are fundamental determinants prevention behavior because they facilitate access to safe transportation vehicle. Promoting prevention and control behaviors in public transportation setting should be adjusted according to population characteristics in pandemic situation [27].

### Limitation and strength

Observation as the method of data gathering was both strength and limitation of our study. On the strength side, observation enabled us to access public behavior in a social setting that was not visible generally. This method gave us the opportunity to provide detailed information targeted with study aims. The limitation of using observation method to collect data was to address the study aims and focus for study samples to record their information ethically that because of the nature of study, it was not possible for researchers.

## Conclusion

Given that social distancing is less observed in the public transportation system in Tabriz, Iran, it is necessary for government to consider and monitor guidelines to protect the passengers from COVID-19 infections by providing measures to maintain physical distance in public transportation systems. It may be possible to force vehicle owners who do not comply with health protocols to keep some distance by imposing fines. Our results suggest that gender, type of vehicle, age, and SES were significant predictors of non-adherence to COVID-19 preventive behaviors in public transport during the pandemic.

## Data Availability

The datasets generated and analyzed during the current study are available from the corresponding author (Leila Jahangiry) on reasonable request.

## Abbreviations

WHO: World Health Organization
UITP: International Association of Public Transportation (from French)
SESs: Socioeconomic Statuses
CVI: content validity index
CVR: Content validity ratio
SPSS: Statistical Package for Social Science
ACE2: Angiotensin-converting enzyme 2
